# Computational Scientific Discovery in Psychology

**DOI:** 10.1177/17456916221091833

**Published:** 2022-08-09

**Authors:** Laura K. Bartlett, Angelo Pirrone, Noman Javed, Fernand Gobet

**Affiliations:** Centre for Philosophy of Natural and Social Science, London School of Economics and Political Science

**Keywords:** computational scientific discovery, AI, creativity, philosophy of science, psychology

## Abstract

Scientific discovery is a driving force for progress involving creative problem-solving processes to further our understanding of the world. The process of scientific discovery has historically been intensive and time-consuming; however, advances in computational power and algorithms have provided an efficient route to make new discoveries. Complex tools using artificial intelligence (AI) can efficiently analyze data as well as generate new hypotheses and theories. Along with AI becoming increasingly prevalent in our daily lives and the services we access, its application to different scientific domains is becoming more widespread. For example, AI has been used for the early detection of medical conditions, identifying treatments and vaccines (e.g., against COVID-19), and predicting protein structure. The application of AI in psychological science has started to become popular. AI can assist in new discoveries both as a tool that allows more freedom to scientists to generate new theories and by making creative discoveries autonomously. Conversely, psychological concepts such as heuristics have refined and improved artificial systems. With such powerful systems, however, there are key ethical and practical issues to consider. This article addresses the current and future directions of computational scientific discovery generally and its applications in psychological science more specifically.

Scientific discovery involves solving complex problems creatively to advance our understanding of the world. The resulting knowledge can then be exploited to treat illness, develop new technologies, and solve critical practical problems. Scientific discovery typically takes a long time—a major discovery often requires intensive and iterative investigations. It is therefore crucial to understand the psychological mechanisms underpinning this behavior and how it could be augmented by technologies such as artificial intelligence (AI). This article first outlines psychological theories of scientific discovery and then argues that AI systems can demonstrate scientific creativity akin to humans, highlighting the ways in which AI has been utilized across different domains to both assist scientists and make new discoveries autonomously. The current and potential implications of computational scientific discovery for research in psychology are then explored, and the positive and negative impacts of using AI in scientific research are considered.

## Scientific Creativity

We define scientific discovery as the generation of new and important theories and the uncovering of phenomena through scientific inquiry, in line with common usage (Gobet et al., 2019). This process is critically important because advancing our knowledge of the world allows us to create new tools, treat illness, and benefit society. A recent example of the importance of scientific discovery for humankind is the design of vaccines against the SARS-CoV-2 virus responsible for the COVID-19 pandemic. To generate new discoveries more efficiently, the mechanisms underpinning creativity and scientific discovery must be understood.

Creativity has intrigued humans since time immemorial and has been a focus of research in psychology for more than a century. Because creativity is subjective and culturally bound, it is hard to define. Although researchers agree that novelty is an essential element of creativity (Weisberg, 2015), the inclusion of other facets such as surprise, success, usefulness, aesthetics, and authenticity vary across the literature (Boden, 1998, 2003; Kharkhurin, 2014; Runco & Jaeger, 2012). A number of psychological theories of scientific creativity have been developed, with psychology of science established as a field of research in its own right (Feist, 2008). Scientific creativity has been conceptualized as a combinatorial process (e.g., Simonton, 1997, 2009), and an extensive field of research has sought to understand the ways in which concepts are combined (e.g., Gabora et al., 2008; Gabora & Steel, 2017; Hampton, 1997). By analyzing 100 important scientific discoveries and 100 inventions, Thagard (2012) provided support for this combinatorial process, suggesting that all of the included discoveries could be accounted for by this framework.

A particularly influential theory posits that creativity can be described as selective search through a problem space (Klahr & Simon, 2001; Newell et al., 1958). Given a starting state, operators are used to move to new states, hopefully reaching a goal state (solution) eventually (Newell & Simon, 1972). Search is made more efficient by the use of heuristics (i.e., rules of thumb) that are likely to be successful. According to this theory, discovering a new scientific law is in essence no different from solving a puzzle such as the tower of Hanoi and could be achieved computationally.

Problem-space theory can be examined in relation to both classic scientific discovery and computational discovery. First, it should be possible to describe established scientific discoveries in terms of search, problem space, and heuristics. Second, it should be possible to replicate famous scientific discoveries in experiments in which the participants are provided with the data used for the discovery. Third, computer programs should be able to replicate or even make new scientific discoveries as they are highly efficient at searching large problem spaces, in particular when they are equipped with domain-specific heuristics. This includes the development of new computational theories in psychology. The following sections briefly review the extent to which these predictions are supported.

## Scientific Discovery

### Observational and laboratory studies of scientific discoveries

The way that scientists carry out research and make discoveries, often after detailed and systematic testing over an extended period of time, has been documented by historians and philosophers of science (e.g., Gillispie, 1960). More recently, scientists who have made important discoveries have been interviewed, and computational models of the processes have been developed (e.g., Karp, 1990). Taking an in vivo approach, Dunbar (1994) observed four research laboratories for 1 year to better understand the cognitive and social processes of scientific discovery. In line with problem-space theory, this analysis revealed similar heuristics across laboratories as well as similarities in fundamental aspects of the cognitive operators used by the scientists, differing mostly in the combinations of these operators. Team dynamics also played a role in the way researchers developed and tested hypotheses, which changed problem representations, indicating the importance of social context in scientific discovery.

Laboratory studies have also been conducted to better understand the cognitive mechanisms underpinning discovery. For example, Schunn and Anderson (1999) contrasted domain-specific experts (psychology faculty doing research in the domain of memory), domain-general experts (psychology faculty doing research not related to memory), and novices (nonpsychology undergraduates) in the design and interpretation of experiments in psychology. They found that domain-specific experts had the best solutions and demonstrated not only domain-specific skills but also many domain-general skills and heuristics. These domain-general skills were also demonstrated by domain-general experts compared with novices, who were missing many of these skills. These results indicate that domain-general skills (such as keeping experiments simple and considering relevant theories when making conclusions) are particularly important for scientific discovery.

### Replicating scientific discoveries

Experimental studies have confirmed that many scientific discoveries of the past can be replicated and reduced to a heuristic search through a problem space, often with common strategies (e.g., Dunbar, 1993; Langley et al., 1987; Qin & Simon, 1990; Zimmerman & Klahr, 2018). For example, Qin and Simon (1990) found that a third of student participants were able to rediscover Kepler’s third law of planetary motion when given the original data. (Note that the variables were not labeled semantically and the data source was unknown.) Likewise, undergraduate students were able to replicate discoveries in the molecular biology of genetic control (Dunbar, 1993), in which successful and unsuccessful answers were distinguished by the goals set by participants. Although creativity is often viewed in society as a mysterious concept, studies such as these support Newell et al.’s (1958) view that it can be explained by mechanisms known to underpin the solving behavior of simple problems such as puzzles.

## Computational Scientific Discovery

If creativity is characterized by searching through a problem space, then computational programs using search should be able to show creativity—and in particular should be able to replicate old and make new scientific discoveries. Indeed, computer systems may do so more efficiently than humans because they can process large amounts of data very quickly.

Computational scientific discovery has a long history (e.g., Langley et al., 1987). For example, Newell et al. (1958) developed the Logic Theorist, which generated proofs for theorems in propositional logic using heuristics. Some of its proofs were more elegant than those proposed by leading mathematicians. This program arguably used a process that resulted in creativity. Indeed, a subfield of computer science (automated theorem proving) has since developed with the aim of verifying mathematical statements mechanically, and proof algorithms are nowadays routinely used for teaching fields of mathematics such as logic (Avigad, 2019). With technological developments, scientists are increasingly using computational processes to solve problems throughout the sciences. Advances in technology can often drive new scientific discoveries, and these discoveries can in turn lead to the development of new technologies (Thagard, 2012).

Genetic programming and neural networks are popular AI techniques being used for scientific discovery. Genetic programming (Koza, 1992) takes a population of programs and evolves them across generations, applying various operations (such as mutating parts of the programs) and finding the best program against a defined fitness function. Such evolutionary algorithms are consistent with the combinatorial nature of scientific discovery. Neural networks, on the other hand, are built of connected artificial units (akin to neurons in biological networks) with particular connection weights in a series of layers—the input layer is activated by an input and the output layer generates a response (Aggarwal, 2018). A system is termed “deep” when it includes a series of hidden layers between the input and output layers.

There are two linked goals in computational scientific-discovery research. The first is to uncover the mechanisms and conditions that led to discoveries by replicating famous examples, and the second is to use this knowledge to engineer AI programs to automate and generate new discoveries.

### Replications

Computational replications of scientific discoveries have been conducted for many years and with increasingly refined computational procedures (e.g., Hakuk & Reich, 2020; Kulkarni & Simon, 1988). KEKADA (Kulkarni & Simon, 1988) is a program that makes theoretical inferences, assesses the acceptability of its knowledge and theories, and models experimental tests. It was able to replicate Krebs’s discovery of the Urea cycle in 1932, a Nobel Prize–winning discovery. Graßhoff and May (1995) extended this research by analyzing available historical documents (notebooks, publications, etc.) for a number of case studies, implementing the common methodological rules that explained all of the studied cases into a computational cognitive model of discovery. The commonalities included heuristics relating to the formation of models and the rules governing the generation and evaluation of causal hypotheses. More recently, Hakuk and Reich (2020) replicated modern discoveries in mechanics (the subfield of physics studying motion) and described a new approach to automated discovery by using the methods and concepts of one discipline to find equivalent knowledge in a different discipline. Consistent with the combinatorial characterization of discovery, this method combined ideas from different domains.

### New discoveries

Advancements in computational power and AI algorithms have led to a number of AI scientific discoveries. AI can be used in research both as a tool to assist humans, allowing researchers more freedom to generate discoveries, and as autonomous discoverers, displaying the creative problem-solving that characterizes scientific discovery and generating new and exciting results that further our knowledge of the world. A full discussion of this literature would cover several volumes, so we focus here on some of the key advances.

One of the earliest systems developed was DENDRAL (Lindsay et al., 1980), which discovered molecular structures by analyzing mass spectrometry data. More recently, the “robot scientist” (King et al., 2004) automated almost the entire scientific process in the domain of functional genomics, an area that had already embraced automation. This system generates hypotheses using observations, designs experiments and runs them, interprets the results in terms of the hypotheses, and then repeats the process. This allows scientists to expedite the process and make creative advances in the field. This instance of automation also serves as an example of where scientific research may be heading, with more automation across all fields.

Computational scientific discovery has led to groundbreaking discoveries in recent years. DeepMind’s AlphaFold is an AI tool that predicts the three-dimensional structure of proteins, solving a 50-year “grand problem” in biology known as the “protein folding problem” (Jumper et al., 2021). To date, this technology has predicted the structure of 350,000 proteins, including 98.5% of human proteins (the structure of only 17% of human proteins had been established experimentally). This in turn advances our understanding of the basic components of cells, leading to the discovery of more efficient drugs to treat illness as well as improved insight regarding gene variations that cause disease in different people. AlphaFold is clearly a powerful and creative tool, reflecting a substantial contribution of AI to scientific discovery. AI has also been used in multiple ways during the COVID-19 pandemic (Abd-Alrazaq et al., 2020), including for the highly efficient development of COVID-19 vaccines (Waltz, 2020), with less than 3 months between the detection of the virus and human trials. Combining messy real-world and experimental data, AI allowed insights and predictions regarding the virus and potential vaccine targets. AI can further assist in processing the large amount of anticipated adverse drug reactions to the vaccine, proving a valuable tool at all stages of this process.

## Computational Discovery and Psychology

Developments in computational and AI systems have far-reaching applications in many domains, and psychology is no exception. AI helps formulate and describe psychological theories. Quite often, theories in the social sciences are verbal, informal, and lack precision; rather than explaining the data, they may be better characterized as a redescription of the data (Addis et al., 2019). Formal computational formulations require the fine details of a theory to be specified—all elements of a theory must be put into a coding language, which involves a detailed description of exactly what is going on. This avoids the use of vague language to describe certain constructs. For example, rather than saying that something is due to procedural memory, this concept is formally defined in a computer program such as ACT-R, along with relevant mechanisms (Anderson et al., 2004).

Although the adoption of AI in psychology is still at an early stage, its use extends into all domains of psychology. In addition to machine learning, which can be used to mine large data files (Dwyer et al., 2018) and evaluate psychological research questions (Elhai & Montag, 2020), AI has led to the development of models and theories, alongside applied uses in clinical psychology. Although psychologists typically focus on explaining human behavior, Yarkoni and Westfall (2017) emphasized the importance of predicting behavior, particularly for applied domains such as clinical psychology. They and others outlined how the prediction of behavior could be achieved by applying ideas and techniques from machine learning such as the analysis of big data and cross-validation (Agrawal et al., 2020), which may address some of the critical problems facing psychology (e.g., the replication crisis and *p*-hacking).

Here, we focus on three examples of AI that benefit psychology: semiautomatic development of theories in psychology, modeling decision-making, and methods for refining clinical diagnostic criteria.

### Semiautomatic development of theories

AI can improve model and theory development in psychological science. Cichy and Kaiser (2019) advocated particularly for the use of deep neural networks (DNNs) in exploratory cognitive science. They suggested that new ideas can come from exploring models; DNNs can serve as proof-of-principle demonstrations, and models can amend and refine fundamental scientific concepts. Agrawal et al. (2020) refined psychological models by comparing them to complex machine-learning models trained on a large data set of moral decisions and produced a theory-based, predictive, and interpretable model of moral decision-making. Genetic programming has been used to effectively describe the interactions of variables in psychometric and lexical access experiments (Westbury et al., 2003). With regard to scientific discovery, Lara-Dammer et al. (2019) demonstrated that a simulated system (NINSUN) emulating human perception based on theories of scientific discovery and perception, is able to make scientific hypotheses in a simple artificial world.

Genetically Evolving Models in Science (GEMS) is a system currently in development designed to automatically generate possible theories of human cognitive behavior (Addis et al., 2019; Frias-Martinez & Gobet, 2007). The system, which extends genetic programming, combines several “operators” (e.g., putting an item into short-term memory or moving covert attention) into a program (i.e., a model) whose predictions are then compared against experimental data from human participants. A population of models, with different combinations of operators, is evolved over a number of generations, with preference given to those most closely matching the human data (for a detailed description of the GEMS system using the delayed-match-to-sample task as an example, see Frias-Martinez & Gobet, 2007). A key benefit of GEMS over other methods is that it does not rely on large data sets. Although this system allows some bias because it requires human input in developing operators, coding the experimental conditions, and setting parameters, this bias is reduced compared with standard theory development. In particular, this system avoids confirmation bias and allows operators from different fields of cognitive psychology that are typically studied separately to be combined. By creating operators relating to these different fields (e.g., memory, attention, and decision-making), novel predictions can be generated that exploit the interplay between these domains. Whereas experts in a given area may fail to appreciate other factors that could influence their topic of interest, GEMS aims to avoid this issue while generating exciting and unexpected theories of human behavior.

In psychology, GEMS has successfully generated scientific theories semiautomatically for a number of experiments (Addis et al., 2016; Frias-Martinez & Gobet, 2007; Lane et al., 2016; Sozou et al., 2017). Although these are initial results that still need to be refined by future research, they establish the validity of the GEMS approach (e.g., Pirrone & Gobet, 2020, 2021).

### Modeling decision-making

Machine-learning techniques have been used to develop and refine theories in decision-making research (Bourgin et al., 2019; Erev et al., 2017; Fudenberg et al., 2022; Noti et al., 2016; Peterson et al., 2021; Peysakhovich & Naecker, 2017; Plonsky et al., 2017). For example, Peterson et al. (2021) collected a large data set on risky-choice decisions in which participants had to choose between different gambles; several machine-learning models of increased complexity were then trained on the data. Each model was based on the literature and included previous (human-generated) theories such as the widely popular subjective utility models and prospect theory. The authors showed that those models could not explain large amounts of variance in the data, even when each model was fine-tuned using the large-scale data set and neural networks that optimized the functional form and the parameters of the model. Interestingly, using machine learning, the authors discovered a new theory of risky choices in which participants adopt sophisticated strategies that are a mixture of previously proposed theories, whose functional form and parameters depend on the specific details of each gamble (such as maximum outcome, minimum outcome, and outcome variability). This new theory and its predictions are likely to drive future studies in the field of risky choices (Plonsky & Erev, 2021) and pave the way for future similar applications in fields across psychology and the cognitive sciences (e.g., Battleday et al., 2020).

### AI in clinical psychology

Alongside model and theory development, AI has the potential to extend the clinical understanding of mental-health conditions, thus allowing the discovery of previously unknown patterns of behavior and a better insight into how different classifications overlap. For example, data-mining techniques have been used to determine which variables can distinguish between groups of high and low suicide risk (Morales et al., 2017). AI can also be used to determine variables that can predict outcome and treatment adherence (Aafjes-van Doorn et al., 2021; D’Alfonso, 2020). AI also has the potential to refine diagnostic criteria (Tai et al., 2019), which could lead to new discoveries and improved knowledge of the factors contributing to different conditions. Short versions of time-intensive self-report measures of personality have been developed using genetic algorithms (Eisenbarth et al., 2015; Yarkoni, 2010), consistent with the original measures across samples, languages, and data-collection methods. This allows researchers to administer such measures more efficiently.

AI-led advances in drug development could drive breakthroughs in clinical research. New scientific discoveries may also be assisted by systems trained to be consultant experts in medical knowledge (e.g., IBM’s Watson). It is likely that automated scientific discovery will have a considerable impact in clinical psychology, especially considering the long history of using formal methods in the domain, for example, to simulate clinicians (Rizzo et al., 2016; Weizenbaum, 1966) and patients (e.g., de Mello & de Souza, 2019; Fitzpatrick et al., 2017; Talbot & Rizzo, 2019; for a review, see Fiske et al., 2019; for a detailed discussion, see Luxton, 2014) and for providing successful clinical diagnoses (Grove et al., 2000).

### Applying psychology to AI development

Although AI has many useful applications in psychology, principles from psychology have long been aiding the development of AI systems (Khetarpal et al., 2020; Lieto & Pozzato, 2020b; Rogers & Mcclelland, 2014; Taylor & Taylor, 2021; for an overview, see Lieto, 2021), which in turn benefits psychological science. For example, the concept of a heuristic, which was central to the success of the Logic Theorist (Newell et al., 1958), was imported from psychology. More recently, the incorporation of human uncertainty (through the collection of an extensive amount of human-categorization data) has improved machine classification (Peterson et al., 2019). Likewise, advances and theories in neuroscience have been applied to AI to improve both the efficiency and accuracy of such systems (for a review, see Hassabis et al., 2017).

The creative combining of concepts that is argued to be a key component of scientific discovery has been modeled by Lieto and Pozzato (2020a) in their typicality based compositional-logic framework. Informed by human cognitive heuristics, this framework makes it possible to automatically generate novel concepts in a human-like way (Chiodino et al., 2020; Lieto et al., 2019, 2021). This framework applies knowledge and theories from cognitive science to overcome problems in artificial systems; correspondingly, the functioning of AI systems allows psychologists to refine their models of cognition. Likewise, with respect to human creativity, understanding the cognitive processes involved can assist in the development of new AI techniques, which in turn can allow psychologists to test theoretical hypotheses regarding creativity and its underlying processes. For example, Gabora and colleagues drew from research on cognitive psychology to develop more refined algorithms that can generate novel and aesthetically appealing artworks (DiPaola et al., 2018; DiPaola & Gabora, 2007). Developments in psychology can inform AI techniques, and these techniques can help generate new discoveries in psychology, highlighting the importance of interdisciplinarity for scientific discovery.

## Computational Scientific Discovery: a Critical Evaluation

The trend toward the automation of the scientific process is important for a number of reasons, not least because of the many new discoveries outlined above. The rate at which data are generated is increasing exponentially; effectively analyzing these data requires the development of tools that excel at efficiently and accurately processing data—tasks for which humans are not well equipped. Because computer systems are optimal for such tasks, they offer significant advantages for scientific discovery. In addition, automating these processes allows human scientists the freedom to engage in higher-level activities, such as theory building, rather than data crunching and manual work. Some see the advancements in AI as a threat to humans. However, because humans and computers excel at different tasks, AI can instead be considered a complementary form of intelligence rather than a replacement (de Mello & de Souza, 2019).

AI is not limited to data processing—it can also produce creative and novel solutions to problems that humans may not have thought of, as illustrated in this article. This is beneficial because there are limits to human creativity and reasoning (Gobet & Sala, 2019), consistent with Simon’s (1956) theory of bounded rationality, which posits that the ability of a decision maker to make a rational choice is constrained by computational capacity and knowledge limitations. However, this forced selectivity can sometimes be advantageous; Gigerenzer and Brighton (2009) argued that heuristics help better manage uncertainty than unbiased, resource-intensive processing. Indeed, machine classification can be improved when human uncertainty is included (Peterson et al., 2019). Knowledge can be detrimental and can lead to a preference for standard responses, even when creative and novel solutions are better (Bilalić et al., 2008). Likewise, living in a society and in a certain context can impose constraints on human creativity; computational creativity is not limited by these constraints, allowing for interesting developments and ideas.

### Ethical considerations

A number of ethical issues arise from using computation for scientific discovery. For example, if controversial theories or patterns in data are found by AI (e.g., using GEMS), the ethical implications must be carefully considered by researchers, particularly the potential negative impacts such discoveries could have. Another example could be taken from clinical psychology: Understanding variables that are predictive of clinical treatment outcomes could be used to minimize barriers for treatment adherence; however, the opposite could also occur, in which patients showing these characteristics are denied treatment. Such unintended uses must be considered and guarded against.

Relatedly, AI-generated discoveries could be a product of bias. Although AI systems are suggested to remove bias, they will likely still reflect the biases of the humans who created them and the data sets they are given. Much psychological research has been criticized as consisting of a limited sample, namely undergraduate psychology students, which is not representative of the general population (Henrich et al., 2010). Although AI can produce novel models of human behavior, these models will be constrained by the nonrepresentative data that they are provided with. This is clearly not an issue limited to AI; however, the power of such technologies and the common view that they are less biased could lead to more reliance on their outputs without considering their inputs.

The impact of biased data has been demonstrated with facial-recognition technology, in which racial and gender bias in data sets resulted in heightened misclassification of women and people of color (Buolamwini & Gebru, 2018). Likewise, AI systems used in the criminal-justice system, from law enforcement to decision-making support (Završnik, 2020), may inherit historical bias—factors that may be used as predictive, such as previous arrests, may be racially biased as a result of systemic discriminatory practices in policing. As outlined by Sgaier et al. (2020), predictive AI can often equate correlation with causation, which can have disastrous consequences—they suggested that causal AI, in which the underlying causes of behavior are modeled, is the essential next step in AI utilization. Bias in AI can also be inherited from using data derived from biased tests; for example, intelligence tests have sometimes been criticized as being culturally biased (Greenfield, 1997; Lozano-Ruiz et al., 2021). Using AI to derive insights regarding intelligence will similarly be subject to the issues surrounding such tests. Although bias exists without it, AI is a powerful tool and so could exacerbate this problem. Similar problems may occur across all areas of AI-generated scientific discovery, and those working with this technology should be aware of these issues.

Although biases can be detrimental for discovery because they can lead scientists to develop and accept incorrect theories, steps can be taken to minimize their influence on AI systems, such as increasing diversity within both the team developing the technology and those testing it. If issues are found with the input data, more and better input can be fed into the systems to rectify this flaw—a much easier process for AI than for humans. Although individual biases are hard to overcome, new approaches and tools can help reduce at least some biases present in AI systems.

The field of AI ethics is very active (for an evaluation, see Hagendorff, 2020) because such a powerful tool can have huge and widespread implications to humans. Although AI systems are generally designed to contribute positively to science and humanity, their unintended negative consequences for some in society and ability to be exploited for damaging purposes must be considered responsibly. To overcome some of these ethical issues, scientists and researchers developing and using such technology should receive training on technological ethics to develop the knowledge and tools for understanding the risks and solving potential ethical issues effectively. Finally, it is important to consider who should be making decisions on whether something is ethical or not. For example, in psychology, experimental research is subject to ethics-committee approval; however, there currently is no parallel in AI.

### Interpretability

To use AI systems for scientific discovery, outputs must be understandable to humans and allow interpretation in the context of current theories and knowledge (Javed & Gobet, 2021). A key issue is that, although the inputs and outputs of an AI system are known, the intermediate steps are sometimes difficult to understand (it is a “black box”), which can affect the interpretability of the output data. Different techniques have been used to address this issue. For example, an artist named Tom White is using AI to generate abstract versions of the images it is trained on to show how AI “sees” the world and demonstrate the underlying algorithms (see https://aiartists.org/tom-white). The system represented various items with abstract blobs, and these blobs were shown to activate the object label more than the real images it was trained on.

Another technique being used is taken directly from psychological science: Psychlab (Leibo et al., 2018) is a resource created to better understand AI systems through classic psychological experiments. Akin to cognitive psychology, it seeks to understand the “cognitions” underlying the outputs given by AI systems and what they are actually doing. Likewise, Taylor and Taylor (2021) recently described the contributions that cognitive psychologists can make in the creation of explainable AI, arguing that the experience of studying the human mind via experimentation is applicable to AI. Although the outputs of AI systems are already leading to great advances in science, it is critically important to understand what is happening in these systems to be able to use the technology for applied purposes.

The importance of interpretability for psychological science has certainly been appreciated, and novel techniques to address this issue are being developed, often drawing from the machine-learning domain. Agrawal et al. (2020) outlined and demonstrated the feasibility of using machine-learning techniques to develop predictive models of psychological phenomena while maintaining interpretability for large data sets through an iterative process they termed “Scientific Regret Minimization”, based on regret minimization in machine learning. Specifically, the fit of a simple psychological model is critiqued against an unconstrained machine-learning model trained on the same data set until the predictions converge. The residuals that may signify novel effects can then be validated in separate experiments. Understanding of the hidden layers of AI systems has also been improved by genetic programming. Evans et al. (2019) and Ferreira et al. (2020) used genetic programming to explain the behavior of a black-box model. Genetic programming does not see the original data but sees the predictions of the black-box model and tries to recreate them. This approach is model-agnostic and can generate explanations for different machine-learning black-box models.

### Clinical considerations

In clinical psychology, the information about a person that some systems gather can be more than a person wants to share. The ability of AI systems, including those aimed at making scientific discoveries, to collect and store such personal data allows for potential breaches of privacy. These systems must be secure to fully protect any sensitive information (Lustgarten et al., 2020), such as the information collected during simulated therapy. Fiske et al. (2019) outlined the following potential practical and ethical issues with using AI in clinical psychology settings: (a) ethical responsibilities related to risk assessment, (b) adherence to codes of practice and duty of care, (c) issues with consent and patients understanding when they are not interacting with a human, and (d) impacts of strong attachments made with AI applications, which are not reciprocal and may reduce real social interaction. Although the use of AI technology in clinical settings presents substantial benefits to well-being, these ethical and practical concerns need to be addressed. Advances in clinical psychology from computational scientific discovery must similarly ensure the safety of those using the systems and the protection of sensitive data.

## Conclusions

AI is a powerful tool that can have a significant role in scientific discovery across all fields of research. It not only allows scientists the freedom to engage in high-level theoretical thinking but also can demonstrate the creativity of autonomously generating novel ideas (e.g., King et al., 2004). The impact that these systems are already having is impressive, and the current trends suggest that they will continue to be more ingrained in the scientific process (Jumper et al., 2021; Peterson et al., 2021). Although creativity has historically been thought of as an unexplainable human concept, evidence from historical records, replications of discoveries, and new discoveries using AI are consistent with creativity and scientific discovery as resulting from problem-solving. Particularly adept at efficiently solving problems, AI can demonstrate creativity and can have a dominant role in generating scientific discoveries in the future. In line with Newell et al. (1958), heuristics obtained through psychological science have been used to improve the efficiency and accuracy of AI, and many AI systems, including AlphaFold, can be described as selectively searching through a problem space. Alongside this, AI systems using evolutionary techniques such as genetic programming depend on random combinations, consistent with the combinatory nature of scientific creativity.

AI can provide innovative ideas that may have taken considerable time for humans, in part because it is less constrained by limits on available knowledge and biases. Scientific breakthroughs are historically the result of extensive experimentation and theorizing and can often be characterized as “thinking outside the box”; this can be achieved much more efficiently by a computational system. The creative advances that artificial systems can provide are critical to accelerating successful scientific inquiry and further pushing our knowledge and understanding of the world.

AI is already proving to be useful for scientific discovery in psychology (e.g., Aafjes-van Doorn et al., 2021; Frias-Martinez & Gobet, 2007; Peterson et al., 2021). It can generate new psychological models of human cognition. The application of concepts from psychology to artificial systems can also constrain models of human behavior. AI can be used as a tool to assist psychologists, for example, in interpreting brain-scan analyses and improving clinical diagnoses. The adoption of such systems in psychology is beginning to grow; however, issues in terms of bias, data protection, interpretability of outputs, and potential unethical uses must be considered.

## Transparency

*Action Editor:* Matthew Rhodes

*Editor:* Laura A. King
